# 6.O. Workshop: Urban design interventions for fostering mental health and mental health literacy

**DOI:** 10.1093/eurpub/ckac129.394

**Published:** 2022-10-25

**Authors:** Organised by: Darmstadt University of Applied Sciences (Germany), Chair persons: Evangelia Chrysikou (EUPHA-URB)

## Abstract

With roughly half of the global population living in cities and an expected rise up to 85 % by 2050, urban environments are central to public health. Yet, they are often perceived as health risk factors due to e.g., noise, pollution, crowding, and anonymity. Indeed, mental disorders show higher incidence in urban contexts compared to rural areas, generally increasing, e.g., due to the Covid19-pandemic as well as to climate change. However, we argue that there is also a rich potential of urban environments to act as resource for mental health and to address mental health issues in the public space. Design interventions such as urban furniture, art and information elements can act as perceived affordances, signifiers and situative stimuli to influence health behaviour, social discourse and individual mindsets regarding mental health. Furthermore, by considering their unique potentials and resources during the design process, the specific surroundings of these interventions can become an integral part of the actual design. As a result, this might change citizens’ perspective on the urban setting as such. While there exists a large body of literature as well as case studies focusing on physical health promotion by urban design interventions, evidence regarding its mental correlate is still scarce. This workshop aims at collecting and elaborating on features in the urban space as potential resources to foster mental health as well as mental health literacy. To that aim, first, three seven-minutes-presentations introduce different disciplinary perspectives on the topic: Starting from a psychological point of view, the relationship between urban environments and mental health will be outlined. Based on insights from human geography, social determinants of urban settings will be elaborated and participatory approaches will be presented as a crucial method for designing effective intervention. Synthesizing these insights, the role of design to influence health behaviour will be discussed and afterwards illustrated using the example of a trail-based design concept as the backdrop for the interactive part. In the second part of this session, first, co-creation methods (such as mindmapping and brainwriting) will be applied together with participants to brainstorm relevant categories, features and potentials of the urban space to foster mental health and mental health literacy. Participants will then be asked to contribute their expertise from their specific field of work particularly with regards to increasing mental health literacy in citizens. This exercise will be highly valuable for participants to acquire interdisciplinary knowledge regarding the influence of urban design interventions and mental health and broaden their perspective and knowledge regarding urban mental health and the built environment. Learnings and variations of this exercise could be also applicable in other settings such as rural communities or semi-public spaces.

**Key messages:**

• This workshop aims at understanding how features of the urban space can be utilised to foster mental health and mental health literacy.

• By co-creatively sharing and elaborating on insights from various disciplines, participants broaden their horizons regarding the interrelations between the urban built environment and mental health.

